# A Case of Gastric Mucormycosis in a 21-Year-Old Patient With Hemophagocytic Lymphohistiocytosis

**DOI:** 10.7759/cureus.32215

**Published:** 2022-12-05

**Authors:** Aysha Noor, Savera Anwar, Hadia Wali, Shayan S Ansari, Zafar Ali

**Affiliations:** 1 Medicine, Shifa Tameer-E-Millat University Shifa College of Medicine, Islamabad, PAK; 2 Medicine, Shifa Tameer-E-Millat University Shifa College of Medicine, islamaabad, PAK; 3 Otolaryngology - Head and Neck Surgery, Shifa International Hospital, Islamabad, PAK; 4 Pathology, Shifa International Hospital, Islamabad, PAK; 5 Pathology, Shifa Tameer-E-Millat University Shifa College of Medicine, Islamabad, PAK

**Keywords:** gastrointestinal mucormycosis, fungal rhino sinusitis, functional endoscopic sinus surgery (fess), gastric biopsy, hemetemesis

## Abstract

Mucormycosis is an angioinvasive, opportunistic infection. Diabetes Mellitus and immunosuppression are the most common risk factors for fungal infection. Without prompt treatment, the infection can be fatal. A 21-year-old male patient presented with gastritis-like symptoms refractory to proton pump inhibitor (PPI) therapy. He recently received treatment for Hemophagocytic Lymphohistiocytosis (HLH), confirmed by bone marrow biopsy and fungal sinusitis. Esophagogastroduodenoscopy (EGD) revealed extensive gastric involvement by Mucormycosis. The patient was given antifungal drugs and a resection of necrotic stomach tissue. Gastric mucormycosis is a rare presentation of the angioinvasive fungus. The patient's young age and lack of distinguishing risk factors such as diabetes or immunosuppression are also unusual. Furthermore, the patient's unique presentation with gastric mucormycosis compounded by a recent diagnosis of Hemophagocytosis lymphohistiocytosis produces a valuable case study in management.

## Introduction

Mucormycosis is a blood vessel-invading fungal infection associated with high morbidity and mortality. This fungus belongs to the class Mucorales. The most common species are Rhizopus, Mucor, Lichtheimia, Rhizomucor, and Cunninghamella [1]. Under a light microscope, mucormycosis appears to have broad, aseptate, or sparsely septate, ribbon-like hyphae. The spores of these organisms are present throughout the environment, especially in the soil and decaying organic matter. Most infections result from the inhalation of fungal spores or bodily entry through disrupted skin [2]. The classification of mucormycosis according to anatomical locations comprises rhinocerebral, pulmonary, cutaneous, gastrointestinal, and disseminated types [1].

Gastrointestinal manifestations are rare. Patients with states of immunosuppression such as neutropenia, corticosteroid use, and solid organ transplantation are the most susceptible. Diabetes mellitus, especially uncontrolled, is another risk factor [3]. Complications of the infection include cavernous sinus thrombosis, disseminated infection, periorbital destruction, osteomyelitis, and death [4]. Mucormycosis diagnosis is based on histological findings or positive cultures from affected sites. According to the European Organization for Research and Treatment of Cancer/Invasive Fungal Infections Cooperative Group (EORTC/MSG) guidelines, recommended management is a combination of treatment with antifungal drugs and surgical resection of the infected lesion [5].

Meanwhile, Hemophagocytic Lymphohistiocytosis (HLH) is an autoimmune disease marked by the proliferation of lymphocytes and macrophages. The impaired immune response cannot protect against infections, increasing infection vulnerability. HLH can either be inherited (primary) or secondary to any severe infection, malignancy, or rheumatologic condition. It has specific diagnostic criteria, including investigations for pancytopenia, hypofibrinogenemia, elevated triglycerides, ferritin, transaminases, and cytokines. Histopathological hallmark is infiltration of the reticuloendothelial system or other organs with lymphocytes and macrophages engaged in hemophagocytosis [6].

Recently, it has been observed that Coronavirus Disease 2019 (COVID-19) patients are more prone to contracting mucormycosis, especially during treatment. Vigilant use of steroids, broad-spectrum antibiotics, and monoclonal antibodies in COVID-19 patients are the cause [7]. The epidemiological distribution of mucormycosis is skewed toward developing countries [8].

## Case presentation

A 21-year-old male from Chakwal, Pakistan, initially presented to a high dependency unit (HDU) with fever and epistaxis for four weeks in early September 2020. Initial complete blood count (CBC) showed pancytopenia, as seen in Table 1. Epistaxis was controlled by nasal packing. The initial differentials included sepsis, dengue infection, human immunodeficiency virus (HIV) infection and COVID-19. Serological tests for HIV, dengue, and COVID-19 were negative. Four days after admission, a bone marrow biopsy conducted to investigate pancytopenia demonstrated hemophagocytosis.

**Table 1:** Complete Blood Count Upon Admission

Test	Value	Reference Range
White Blood Cell Count	3260 /uL	4000 - 10500 /uL
Red Blood Cell Count	3.85 m/uL	4.5 - 6.5 m/uL
Hemoglobin	11.5 g/dL	13.5 - 18.0 g/dL
Hematocrit	32.5 %	42.0 - 52.0 %
Mean Corpuscular Volume	84.4 fL	78.0 - 100.0 fL
Mean Corpuscular Hemoglobin	29.9 pg	27.0 - 31.0 pg
Mean Corpuscular Hemoglobin Concentration	35.4 g/dL	32.0 - 36.0 g/dL
Platelet Count	24000 /uL	150,000-400,000 /uL
Absolute Neutrophil Count	1499 /uL	
Neutrophil	46 %	54 - 62 %
Lymphocyte	42 %	25 - 33 %
Monocyte	12 %	1 - 4 %
Eosinophil	0 %	1 - 3 %
Basophil	0 %	0 - 0.75 %
Red Cell Distribution Width	15.4 %	11.5 - 14.0 %
Mean Platelet Volume	12.0 fL	6.8 - 10.2 fL

Upon further investigation, the patient was discovered to fulfill the HLH criteria, as seen in Table 2. The labs demonstrated elevated ferritin, raised triglycerides, liver dysfunction, disseminated intravascular coagulation (DIC), and pancytopenia. Furthermore, the patient's Epstein-Barr virus (EBV) viral load was positive. Therefore, the patient was diagnosed with HLH secondary to EBV.

**Table 2:** The Hemophagocytic Lymphohistiocytosis (HLH) criteria workup.

Test	Value	Reference Range
Alanine Transaminase	609 IU/L	5 - 40 IU/L
Total Bilirubin	3.2 mg/dL	0.3 - 1.7 mg/dL
Ferritin	5485 ng/mL	30 - 400 ng/mL
Fibrinogen	84.0 mg/dL	199.0 - 463.0 mg/dL
Activated Partial Thromboplastin Time	49 sec	24.8 - 36.2 sec
Prothrombin time	13.10 sec	9.5 - 11.7 sec
Triglycerides	320 mg/dL	Normal < 150 mg/dL Borderline High 150 - 190 mg/dL
D-Dimers	19.11 mg/L	≤ 0.50 mg/L

**Figure 1:**
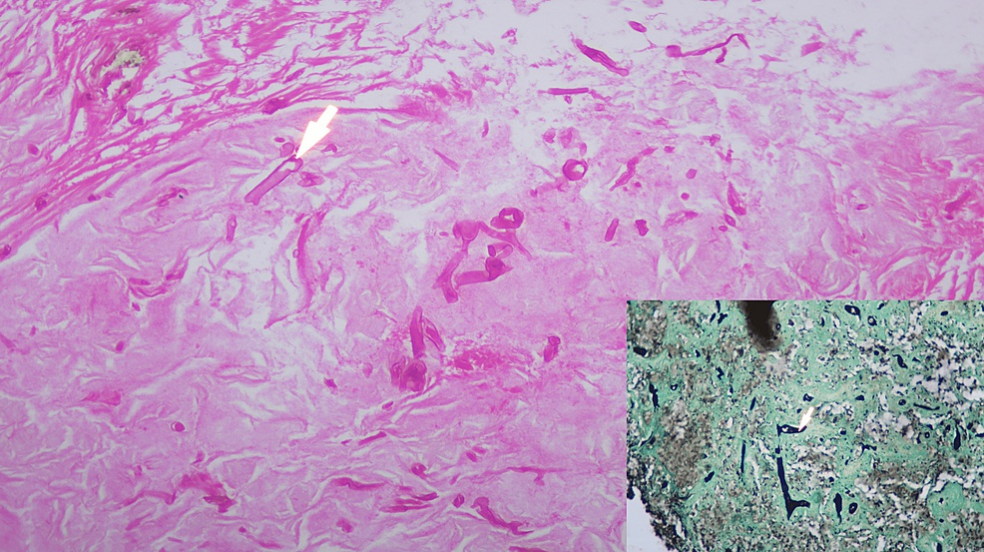
Photomicrograph showing broad aseptate fungal hyphae of Mucormycosis species of paranasal sinus mucosa. Inset showing GMS special stain highlighting the fungal elements.

HLH 94 protocol was initiated immediately. Intrathecal chemotherapy was administered by injecting hydrocortisone succinate 15 micrograms and methotrexate 12mg. The patient responded well to treatment, as demonstrated by the resolution of DIC, pancytopenia, and fever. Due to the patient's complaints of persistent headaches, a brain MRI was conducted, which revealed sinusitis. Consequently, on the 29th day of admission, i.e., on 29th September 2020, Functional Endoscopic Sinus Surgery (FESS) was performed for tissue sampling, and the potassium hydroxide (KOH) of the samples confirmed the growth of Rhizopus and Aspergillus Flavus as seen in figure 1.

With only two weeks of completion, the HLH 94 protocol was halted due to the diagnosis of mucormycosis in the right maxillary sinus. Antifungal therapy commenced with four doses of isavuconazole and liposomal amphotericin B for three days, followed by isavuconazonium for seven days and then posaconazole for two weeks.

Since his hospital admission in early September, the patient had complained of gastritis-like symptoms, which were refractory to treatment with proton pump inhibitors (PPIs). Due to the recent diagnosis of fungal sinusitis, there was a high suspicion of Mucormycosis infection. Therefore, an Esophagogastroduodenoscopy (EGD) was performed, revealing extensive stomach involvement by Mucormycosis involving the posterior wall and greater curvature of the stomach. He was given amphotericin B for two weeks to salvage the stomach and discharged. Ten days after the initiation of treatment, i.e., on 5th October 2020, the patient arrived at the Emergency Room (ER) with massive gastrointestinal (GI) bleeding. He was taken to the operating room immediately. A large ulcer was visualized, and a near subtotal gastrectomy was conducted, which left a small gastric pouch approximately 4 cm from the gastroesophageal junction. A ROUX en-Y loop was formed measuring 5 cm from the duodenojejunal flexure and anastomosed with the stomach pouch. The previous antifungal regime was continued with the addition of monthly Vitamin B12 injections.

The patient complained of facial pain, nausea, and vomiting on follow-up. A CT scan nose and paranasal sinuses were performed on the 36th day of the first FESS (November 2020), which showed persistent disease of the sinuses with bony erosion involving the skull base. On the 38th day, re-FESS was performed for tissue sampling and clearing the sinuses. Tissue culture and KOH of the samples again showed growth of fungus. Afterward, antifungal therapy continued with an injection of liposomal amphotericin 150 mg intravenously every 2 hours for three weeks. On day 20 of liposomal amphotericin, the patient's facial pain, nausea, and vomiting symptoms resolved; therefore, antifungal therapy was ceased.

A follow-up CT scan was ordered on the 66th day of the first FESS, i.e., on 24th January 2021. A repeat FESS procedure was conducted to take additional samples, and repeat KOH testing did not show the growth of fungal species. 

After the patient's fungal sinusitis resolved, he has been stable, and no episodes of pancytopenia, pyrexia of unknown origin, or DIC have been recorded. The patient continues to have regular follow-ups every two months for close monitoring of his HLH disease, during which a history of symptoms, general examinations, and investigations are performed, all of which have been reassuring thus far. 

## Discussion

Gastric mucormycosis is an infrequent manifestation of mucormycosis infection. The stomach is most commonly involved among those infected, followed by the colon, small intestine, and esophagus [9]. The primary mode of Mucorales entry into the gastrointestinal tract is via ingestion of contaminated food or moldy bread. However, it can also be due to contaminated healthcare devices such as tongue depressors. The angioinvasive fungal disease causes necrotizing vasculitis, leading to thrombosis and tissue infarction [10].

Uncontrolled diabetes mellitus and severe immunodeficiencies, especially hematological malignancies, are significant risk factors for mucormycosis [9]. Previous case reports of gastric mucormycosis describe patients with a history of heroin abuse and chronic alcoholism in addition to diabetes [11,12]. Furthermore, another study reported a case of gastric mucormycosis in a patient undergoing chemotherapy for a Stage 1 germ cell tumor of the mediastinum [13]. Most gastric mucormycosis case reports describe middle-aged patients, and the early to mid-twenties seem to be an uncommon age for developing gastric mucormycosis.

In contrast, Ahmed et al. reported gastric mucormycosis in two infants and one patient who was 23 years old, like our patient [14]. Hemophagocytic Lymphohistiocytosis (HLH) diagnosis qualified our patient for the HLH 94 protocol treatment. The immunosuppressive therapy compounded by the HLH disease process may have contributed to the patient's immunocompromised state. Thus, increasing the risk of developing invasive mucormycosis. 

Presentations of gastric mucormycosis vary from patient to patient. The clinical manifestations can range from hematemesis to acute epigastric pain to generalized peritonitis [11-13]. Our patient complained of gastritis-like symptoms that were refractory to proton-pump inhibitors. The subsequent month, the patient presented in the ER with a massive GI bleed.

According to EORTC/MSG guidelines, the probability of invasive fungal infections involves three levels of diagnosis: proven, probable, and possible. A proven diagnosis consists of fungus detected on histological analysis or culture of a tissue specimen taken from a disease site. The probable category consists of host factors and clinical criteria. Severe immunodeficiency is considered a host factor, such as HLH. Furthermore, a clinical criterion is a sinonasal infection confirmed by imaging, such as a nasal ulcer with black eschar.

Additionally, a central nervous system (CNS) infection detected by focal lesions on imaging or meningeal enhancement on MRI or CT must be present [5]. Modalities of diagnosis for mucormycosis include routine blood work, biopsy, culture, and imaging [15]. Tissue samples of mucormycosis, obtained through exploratory laparotomy, stain excellently with hematoxylin and eosin sections. The characteristic granulomatous inflammation seen in histopathological specimens will be absent in immunosuppressed individuals. Culture media most often used for Mucormycosis species include Sabouraud agar and potato dextrose agar [16]. In recent years, scientists have been using molecular-based techniques such as polymerase chain reaction (PCR), restriction fragment length polymorphism (RFLP), and DNA sequencing of defined gene regions to diagnose mucormycosis with success [3]. Various anatomical manifestations of mucormycosis must be diagnosed using site-specific techniques. For example, investigating gastrointestinal mucormycosis requires endoscopic procedures demonstrating characteristic fungal or necrotic lesions [10].

The patient's existing HLH disease compounded his antifungal treatment. The HLH was treated according to the HLH 94 protocol, consisting of initial intensive therapy with immunosuppressive and cytotoxic agents for eight weeks [6]. The protocol aims to induce remission of the disease activity. Unfortunately, due to the severe invasive fungal infection of the patient, immunosuppressive therapy became contraindicated. Furthermore, Arena et al. propose that the causative organism should be treated first in cases where an HLH patient has a fungal infection [9].

Successful treatment of mucormycosis depends on early diagnosis and a multimodal approach. The treatment involves the discontinuation of any predisposing factors as well as the administration of intravenous antifungals at optimal doses [10]. Liposomal amphotericin B is the main antifungal used for initial management [10]. Published case reports about gastric mucormycosis agree that the mainstay of treatment is the resection of infected and necrotic tissue and antifungal therapy as soon as possible [9-14]. Mucorales hyphae branching is extensive; therefore, healthy tissue is sacrificed to prevent a recurrence.

## Conclusions

Mucormycosis is a highly fatal and invasive fungal infection. Patients with uncontrolled diabetes or severe immunodeficiencies such as HLH are most at risk. Presentation of the disease ranges from gastritis-like pain to hematemesis and massive GI bleeding. The outcome of Gastrointestinal fungal infection is highly dependent on early diagnosis and treatment. Therefore in patients with apparent risk factors and unusual gastric complaints, fungal infection, specially mucormycosis, should be kept in the patient's differential diagnosis. Secondly, if a patient is diagnosed with gastric mucormycosis, early surgical intervention and antifungal therapy, including liposomal amphotericin B, should be started without any delay to save the patient's life.
